# Healthy cats tolerate long-term daily feeding of Cannabidiol

**DOI:** 10.3389/fvets.2023.1324622

**Published:** 2024-01-24

**Authors:** Jennifer C. Coltherd, Robyn Bednall, Anne Marie Bakke, Zack Ellerby, Christopher Newman, Phillip Watson, Darren W. Logan, Lucy J. Holcombe

**Affiliations:** WALTHAM Petcare Science Institute, Waltham-on-the-Wolds, Melton Mowbray, United Kingdom

**Keywords:** cannabidiol, cat, safety, CBD, cannabinoids, feline

## Abstract

Cannabidiol (CBD)-containing products are widely commercially available for companion animals, mirroring popularity in human use. Although data on the safety and efficacy of long-term oral supplementation are increasing in dogs, evidence remains lacking in cats. The purpose of these studies was to address gaps in the knowledge around the long-term suitability and tolerance of a tetrahydrocannabinol (THC)-free CBD distillate in clinically healthy cats. The studies were randomized, blinded, and placebo-controlled. The first study supplemented cats with either a placebo oil (*n* = 10) or with 4 mg/kg body weight (BW) CBD in placebo oil (*n* = 9) daily, with a meal, for 4 weeks. The concentration of CBD in plasma was measured over 4 h at d0 (first dose) and again at d14 (after 2 weeks of daily dosing). The second study supplemented cats daily with either placebo oil (*n* = 10) or 4 mg/kg BW CBD in placebo oil (*n* = 10) for a period of 26 weeks. A comprehensive suite of physiological health measures was performed throughout the study at baseline (week 0) and after 4, 10, 18, and 26 weeks of feeding, followed by a 4-week washout sample (week 30). Postprandial plasma CBD time course data, at both d0 and d14, showed a peak plasma CBD concentration at 2 h after the dose. This peak was 251 (95% CI: 108.7, 393.4) and 431 (95% CI, 288.7, 573.4) ng/mL CBD at d0 and d14, respectively, and the area under the curve concentration was higher by 91.5 (95% CI, 33.1, 149.9) ng-h/mL after 2 weeks of supplementation (*p* = 0.002). While in the first study the CBD group displayed increased alanine aminotransferase (ALT; 68.7 (95% CI, 43.23, 109.2) U/L) at week 4 compared to the placebo control group [1.44-fold increase (95% CI, 0.813, 2.54)], statistical equivalence (at 2-fold limits) was found for ALT across the duration of the second, long-term study. All other biochemistry and hematology data showed no clinically significant differences between supplement groups. Data presented here suggest that a THC-free, CBD distillate fed at a dose of 4 mg/kg BW was absorbed into plasma and well tolerated by healthy cats when supplemented over a period of 26 weeks.

## 1 Introduction

*Cannabis sativa*, also known as hemp, contains hundreds of phytocompounds including cannabidiol (CBD), tetrahydrocannabinol (THC), cannabidiolic acid (CBDA), cannabigerolic acid (CBGA), and cannabivarin (CBDV) to name a few (1, 2). These compounds differ in their chemical properties and physiological impacts. CBD is the non-psychotropic, and main, component of *C. sativa*, receiving a wealth of interest over recent years due to its potential for anti-inflammatory, anti-oxidative, neuroprotective, and anti-anxiety effects (3). As such it has made for a promising candidate in many therapeutic areas such as pain management in osteoarthritis, epilepsy, Alzheimer’s disease, multiple sclerosis, and anxiety in humans (3), benefits which may translate to animals (4). CBD, therefore, shows efficacy for a wide variety of conditions which act via numerous pathways linked to the endocannabinoid system (5), and these have been collectively labeled the endocannabinoidome (6), indicating there are several potential modes of action. These include, but are not limited to, G-protein-coupled cannabinoid receptors type 1 and 2 (CB1 and CB2), transient receptor vanilloid type-1 (TRPV1) channel, G-protein-coupled receptor 55 (GPR55) or 119 (GPR119), and peroxisome proliferator-activated receptors (PPAR)α and γ (5). There are also promising effects of CBD in the treatment of neurodevelopmental disorders such as schizophrenia directly and indirectly via dopamine receptors (7). THC, a psychoactive component of *C. sativa*, is found in small quantities (lower than 0.3%) in hemp extracts (3). When present in combination with CBD during companion animal trials, THC is thought to lead to the observation of more severe dose-dependent adverse events (8).

To date, no regulatory body has deemed the current safety and efficacy literature surrounding CBD sufficient for pets (9). Despite this, the use of CBD products in pets has increased as they have gained traction in the human market (10). Recent publications have demonstrated that 4 mg CBD/kg BW per day administered over a 6-month period in dogs is well tolerated (11) and that a single 4 mg/kg BW dose reduces anxiety during a car journey or separation test (12). Another study evaluating the effect of long-term supplementation of a CBD-rich dose in beagles found that it was generally well tolerated (13). However, due to higher frequency of abnormal fecal scores and a higher alkaline phosphatase (ALP), the authors advised extra caution at a daily 10 mg/kg BW dose compared to 5 mg/kg BW (13). In contrast, very little information exists on the safety of CBD for cats, and there is no current literature on its efficacy in the treatment of disorders.

In one feline CBD tolerance study, eight cats were fed capsules containing 2 mg/kg CBD in fish oil (50:50 mix of CBD and CBDA) twice a day for 12 weeks (14). All biochemistry data were found to be within normal ranges with the exception of one cat which had elevated alanine aminotransferase (ALT) during the treatment, and no further information on health of the cat was provided. The authors heavily caveated that the lack of a control group limited the ability to know whether any of these effects were due to the CBD dose, the carrier oil, or other environmental factors (14). Pharmacokinetic data from the same manuscript established that CBD could be detected in serum for up to 8 h. In another recently published study, CBD pharmacokinetics showed a mean peak CBD value of 282 mg/mL at 2 h following a CBD dose of 1.37 mg/kg (15). These cats were dosed twice a day with a paste comprised of mainly CBD and CBDA (6.4 mg/g and 5.3 mg/g, respectively), with THC, THCA, CBG, and CBGA included at 25-fold lower amounts, and meals were fed 1 h after dosing (15). When comparing dog and cat, data suggest that CBD has a lower bioavailability in cats compared to dogs but with a similar half-life (16). Although food is known to increase bioavailability of CBD in humans (17), there have been no postprandial investigations of CBD distillate given in low doses concurrently with a meal in cats to understand whether this finding is translatable. The CBD-containing anti-seizure drug Epidiolex^®^, when given to fasted and fed cats in a cross-over design study at a dose of 5 mg/kg BW, identified a higher area under the curve and maximum concentration of CBD in the plasma of fed cats (18).

Here, we describe the findings of a 6-month tolerance study of a single daily 4 mg/kg dose of a THC-free, CBD distillate and an additional four-week postprandial plasma CBD time course study in healthy adult cats.

## 2 Materials and methods

### 2.1 Animals and husbandry

Two studies were reviewed and approved by the Waltham Animal Welfare and Ethical Review Body and conducted under the authority of the Animals (Scientific Procedures) Act 1986. To ensure suitability for the study, the cats underwent a pre-study health assessment, including a physical examination by a registered Veterinary Surgeon and hematological, plasma biochemical, and urine analysis to confirm the absence of underlying conditions. Cats were housed at the Waltham Petcare Science Institute, grouped in social rooms under routine husbandry conditions and were extensively trained for and habituated to all procedures. Cats were observed within these social rooms for feces and free-catch urine collections. For all blood samples, cats were given topical local anesthesia (1 mL EMLA™ cream 5%; AstraZeneca) prior to either jugular or cephalic blood draws. Where a cephalic sample was obtained, a 22G catheter was positioned, which remained in place for the duration of the sampling period. On the day of sampling, cats were allowed to return to their social rooms and monitored closely for any welfare concerns (i.e., pulling out the catheter or scratching at the jugular area). Cats’ health was monitored via weekly (study one: postprandial plasma CBD time course) and fortnightly (study two: tolerance test) physical health and biochemistry, hematology, and urinalysis data reviews with Veterinary Surgeons who were blinded to the groups. Throughout the study, commercial single batch (Royal Canin^®^ Instinctive wet and Royal Canin^®^ Fit-32 dry format) diets were offered in amounts required to maintain an ideal body weight (BW) and body condition score (BCS), assessed according to a 9-point scale. These were used to calculate individual MER (19). The diets underwent nutrient analysis (Eurofins, United Kingdom), and both were confirmed as complete and balanced according to minimum requirements set by the Association of American Feed Control Officials (AAFCO). Water was available *ad libitum*.

Study one, for postprandial plasma CBD time course: 19 healthy adult cats took part in a 4-week study (8 female cats and 11 male cats, age 1.4 to 10.1 years, and weight between 3.42 kg and 5.58 kg).

Study two, to assess long-term tolerance: 20 healthy adult cats took part in a 26-week study, (6 female cats and 14 male cats, age 2.1 to 10.8 years, and weight between 3.39 kg and 5.77 kg). Sixteen of these 20 cats had previously participated in the pharmacokinetic profiling study, with a washout of 9 weeks between studies.

### 2.2 CBD description and dosing

Hemp-derived distillate and placebo oil were acquired from Kazmira LLC (Colorado, United States). The CBD oil was analyzed by a third-party laboratory for full-spectrum analysis of cannabinoid content (including CBD and THC), potential contaminants, and potency (Botanacor Laboratories, Colorado, United States). The THC content was below the limit of analytical detection (<0.02 mg/mL), and no other cannabinoids were detected except for trace amounts (estimated at 0.17 mg/mL) of cannabidivarin, below the limit of quantification (0.32 mg/mL). The distillate was diluted with a food-grade sunflower oil and flavored with 1% rotisserie chicken type, natural flavor blend (Apex Flavors, Inc. Maryland, United States) to provide CBD at a final concentration of 43.76 mg/mL. The placebo oil was the food-grade sunflower oil with 1% rotisserie chicken type, natural flavor blend (Apex Flavors, Inc. Maryland, United States). Each cat was provided with 8 g “bolus” of a commercial pate (Purina^®^ Gourmet Gold) food with the supplement incorporated to provide a dose of 4 mg/kg BW (the placebo oil amount was calculated as if it was the concentration of the CBD oil). The bolus was offered once a day, prior to the morning meal, and consumption was recorded and monitored.

### 2.3 Study design

Both studies were blinded. Cats were randomized and balanced across two parallel treatment groups: CBD and placebo. When balancing the groups, age, sex, and housing location were considered. The cats were then split into two staggers for logistical ease (10 cats per stagger group, 4–6 cats in each treatment group), with a 1-week offset between stagger groups for trial initiation and collection of samples. To accurately dose CBD, cats were weighed weekly.

Cats were fed in a wet food am, dry food pm feeding regimen for 4 weeks before a baseline blood sample was collected for each study, and this feeding pattern was then continued for the duration of the study.

#### 2.3.1 Study one: postprandial plasma CBD time course

Following the collection of an overnight fasted (>14 h) blood sample (max 4.1 mL) on day 0 (first CBD dose) and day 14 (after 2 weeks of daily supplementation), the cats were orally dosed with their CBD or placebo oil, mixed with 1 mL of Sheba^®^ creamy snack (now called Dreamies^®^ Creamy) from a needle-less syringe. These were willingly consumed, and the full dose was administered before the rest of the samples were collected to determine CBD concentrations in the plasma at 1 h, 2 h, and 4 h post-CBD dose and morning meal. On all other days, cats were offered the pate bolus with supplement. Fasted samples were also collected at week 4 without further postprandial sampling.

#### 2.3.2 Study two: long-term tolerance test

Overnight fasted (>14 h) blood samples (max 4.8 mL) were collected at weeks 0, 4, 10, 18, and 26. An additional blood sample (week 30) was collected 4 weeks after supplementation was ceased. A blood sample was collected for a veterinary health check at week 2, and this was not analyzed as part of the trial data set. For logistical reasons, feces and urine samples were collected between 3 and 9 days after blood sampling (at weeks 0, 10, 18, and 26) for urinalysis (urine only) and CBD analysis.

### 2.4 Measures and analyses

#### 2.4.1 Blood-based measurements

Lithium heparin-treated blood was centrifuged at 2,000 g, and the resulting plasma was used for the determination of standard biochemistry parameters: total protein, albumin, inorganic phosphate, alkaline phosphatase (ALP), alanine aminotransferase (ALT), aspartate aminotransferase (AST), calcium, cholesterol, urea, creatinine, triglycerides, magnesium, sodium, potassium, chloride, and glucose, using an AU480 analyzer (Beckman Coulter, United States). EDTA-treated blood was collected for the measurement of standard hematology parameters using a three-part differential automated hematology analyzer (IDEXX ProCyte Dx, Buckinghamshire, United Kingdom). Parameters measured were total leukocyte count, differentiated leukocyte counts as a number and percentage (neutrophils, eosinophils, basophils, lymphocytes, and monocytes), total erythrocyte count, hemoglobin concentration, hematocrit, mean corpuscular volume, mean corpuscular hemoglobin, mean corpuscular hemoglobin concentration, erythrocyte distribution width, platelet count, and mean platelet volume. EDTA-treated blood was also collected for CBD quantification. Serum clot activated blood was centrifuged and stored at 4°C before analysis by IDEXX Laboratories (United Kingdom), and total bilirubin, gamma-glutamyl transferase (GGT), and fasted bile acids were measured using an AU5800 clinical chemistry analyzer (Beckman Coulter; United States). Additionally at baseline and 26 and 30 weeks, serum clot activated blood was used to evaluate markers of bone turnover: bone-specific alkaline phosphatase (BALP) and carboxy-terminal telopeptide cross-links (CTx) using MicroVue™ BALP ELISA kit (Quidel^®^, United States) and Serum CrossLaps CTX-I ELISA kit (Immunodiagnostic Systems Limited; United Kingdom), respectively. Both assays were performed according to the manufacturer’s instructions on a Synergy HT plate reader (Agilent Technologies, United States) and have sensitivity limits of 0.7 U/L for BALP and 0.02 ng/mL CTX. Reference ranges, where shown, refer to those published by Antech^®^ Diagnostic Laboratories for biochemistry analysis and IDEXX Laboratories (United Kingdom) for complete blood count and liver panel (Bilirubin, GGT and Bile Acids) data.

#### 2.4.2 Urinalysis

Urine was collected (min 3 mL total volume) using a free-catch method with a uripet (Fisher Scientific; United Kingdom), 1 week after the blood sample timepoints, i.e., at weeks 1, 11, 19, and 27. Urine-specific gravity was measured using a refractometer (J.A.K. Marketing Ltd., United Kingdom), and glucose, bilirubin, ketone, specific gravity, blood, pH, protein, urobilinogen, nitrite, and leukocytes were analyzed using the Status Plus Analyzer with Multistix^®^ 10SG urine test strips (Siemens Healthcare Limited; United Kingdom). An aliquot of urine was also processed for CBD quantification.

### 2.5 Feces collection

A single fresh fecal sample was collected alongside urine at weeks 1, 11, 19, and 27, a core sample was obtained, and CBD levels were quantified.

### 2.6 CBD extraction and mass spectrometry analysis

Extraction and analysis of CBD in samples were adapted from the method used by Vaughn et al. (20), fully validated in-house (11). In brief, for urine samples, the internal standard (300 μL of 40 ng/mL, CBD-d3) was aliquoted into 100 μL of feline urine and vortexed for 5 s. Samples were centrifuged (13,201 g for 5 min), and 325 μL of the supernatant was aliquoted into a labeled glass amber vial containing 650 μL 0.1% formic acid in water. The sample was vortexed again (5 s) and analyzed as described. For feline fecal samples, the internal standard (750 μL of 300 ng/mL, CBD-d3) was aliquoted into microfuge tubes containing 0.25 g (±0.01 g) of feces and vortexed for 30 min. Samples were centrifuged (2,292 g for 10 min), and 390 μL of the supernatant was aliquoted into a fresh tube containing 780 μL of 0.1% formic acid in water and vortexed (5 s) and centrifuged for a second time (17,968 g for 10 min). The supernatant was aliquoted to a labeled glass amber vial, vortexed again (5 s), and analyzed as described. For plasma, the internal standard (60 μL of 40 ng/mL, CBD-d3) was aliquoted into 20 μL of feline plasma and vortexed for 5 s. Samples were centrifuged (13,201 g for 5 min), and 65 μL of the supernatant was aliquoted into a labeled glass amber vial containing 130 μL 0.1% formic acid in water. The sample was vortexed again (5 s) and analyzed as described.

A liquid chromatograph coupled with triple quadrupole mass spectrometer (Agilent 6460C LC-QQQ-MS, Agilent, United States) was used for analysis. A Kinetex 2.6 μm Phenyl-Hexyl 100A, 50 × 2.1 mm column was used in conjunction with an X3 SecurityGuard ULTRA Cartridge ultra-high performance liquid chromatography (UHPLC) phenyl column guard (Phenomenex, Cheshire, United Kingdom). The mobile phase was delivered at a flow rate of 0.4 mL/min, and the gradient parameters were as follows (solvent A was 0.1% formic acid in ultra-high quality (UHQ) water, and solvent B was 0.1% formic acid in acetonitrile): 0 min: 30% B, 5.3 min: 95% B, 6.3 min: 70% B. The scanning conditions were in multiple reaction monitoring (MRM) mode. Cannabidiol (CBD) and Cannabidiol-D3 (CBD-d3, used as an internal standard) certified reference materials were obtained from Fisher (Loughborough, United Kingdom). Samples were analyzed against a set of 10 linearity standards between 0.25 and 2,000 ng/mL CBD, each prepared with CBD-d3 to a final concentration of 10 ng/mL.

### 2.7 Statistical analysis

#### 2.7.1 Power analysis

The sample size for this study was determined through *a priori* power analysis by simulation, for the primary measure of ALT. Adult cat ALT measurements from historical data sets were used to estimate the within- and between-cat variance components. Using these variance components, data sets were simulated in the design described for the tolerance study (parallel with 2 treatments, 6 timepoints including baseline) for a range of cat numbers. For each cohort size, 1,000 data sets were simulated, and the analysis and planned comparisons described below were applied to each. The power was calculated as the percentage of the 1,000 data sets where equivalence could be declared at 2-fold limits, using a significance level of 5%, given that no difference between the treatment groups or timepoints had been induced.

The estimated sample size required to achieve 80% power was 12 cats (6 per treatment group). Given the nature of the study and potential for study fatigue, the total sample size was inflated to 20 cats (10 per treatment group). This powering was used for both study 1 and study 2.

#### 2.7.2 Alanine aminotransferase

For ALT, a linear mixed model was fit to the log10 concentration, with treatment group, timepoint (i.e., weeks on trial), and their interaction as categorical fixed effects, and a random intercept for animal. Within each treatment group, comparisons between baseline and each subsequent timepoint were tested, and at each timepoint, a comparison between treatment groups was also tested. All comparisons were tested for equivalence at 2-fold limits using two one-sided tests (TOSTs) at a 5% significance level, adjusted for family-wise error-rate (FWER; using the ‘single-step’ method of the R package “multcomp” implemented through the glht function). Note that FWER adjustment was made according to the number of contrasts performed, where contrasts are defined as each pair of TOSTs, due to the requirement that both tests were significant to infer equivalence. Significant *p*-values for the tests are reported, alongside the back-transformed estimates of the difference (i.e., fold changes) with 95% confidence intervals in each case. Back-transformed estimates of the mean and FWE-corrected 95% confidence intervals are also provided for each treatment/timepoint.

#### 2.7.3 Secondary measures

For secondary measures, excluding those with insufficient samples (LIH, GGT, PDW, P_LCR) or insufficient sample variability (BCS), linear mixed models were fit with the same fixed and random effect structures as for ALT. Assumptions of normality were assessed through visual inspection of residuals, and, if this assumption was deemed to be violated, the response variable was log10 transformed. Pairwise planned comparisons between groups at each timepoint and between baseline and each subsequent timepoint for each group were tested for differences at a 5% significance level, with multiplicity correction (FWE, ‘single-step’) applied within but not across models. Significant *p*-values are reported alongside the corresponding difference estimates and 95% confidence intervals in each case. Estimates of the mean and 95% confidence intervals are also provided for each treatment/timepoint. Fasted CBD in plasma, for the CBD group weeks 2 to 26, was modeled with a sole categorical fixed effect of timepoint, a random intercept for animal, plus the incorporation of variance weights by timepoint, due to anticipated differences in plasma CBD variability over time. All values below LOD were imputed. Means and adjusted 95% confidence intervals are reported for modeled data, alongside raw data for all groups and timepoints. Pairwise contrasts are not reported.

#### 2.7.4 CBD concentrations in blood plasma and AUC

Both CBD concentrations in blood plasma (at each timepoint) and area under the curve (AUC calculated per hour and over all 4 h from ingestion) were analyzed as further outcome variables. AUC was calculated using a linear trapezoidal method though custom code implemented in R:


$$AUC=\frac{1}{2}\left(C_{1}C_{2}\right)\left(t_{2}-t_{1}\right)$$

Due to measurements for the placebo group being unanimously below the LOD, data for this group were excluded from these analyses. For AUC, a linear mixed model was fit to data for CBD treatment group only, untransformed, with timepoint (i.e., Weeks 0 vs. 2) as the only fixed effect, and a random intercept for animal. For raw CBD concentration, a linear mixed model was again fit to data for the CBD treatment group only, untransformed, with timepoint (i.e., Week 0 vs. 2), hour (0 vs. 1 vs. 2 vs. 4, categorically coded), and their two-way interaction as fixed effects, plus a random intercept for animal. For the raw CBD model, variance weighting was applied (using the R package ‘nlme’) due to heteroscedasticity between timepoints (i.e., variance was much lower at hour 0). Pairwise comparisons between timepoints are reported, and FWE-corrected 95% CIs are plotted for visual comparison against the LOD (12.0).

#### 2.7.5 Statistical software and packages

All analyses were performed in R version 4.2.2 (2022-10-31), the R Foundation for Statistical Computing (21). Packages necessary for analysis were lme4 (22), nlme (23), and multcomp (24).

## 3 Results

### 3.1 Postprandial plasma CBD time course study

#### 3.1.1 Observations from study

At the 4-week timepoint, three cats were noted as having unusual responses. One cat had blood in urine with no other signs of illness, one cat had asymptomatic high ALT and AST, and the third cat had inappetence, pyrexia, and high ALT and AST. Both cats showing increased ALT and AST were in the CBD group and were subsequently removed from consideration for the long-term study. Veterinary consultation determined the presence of infection in the pyrexic cat. Due to identification of a likely treatment-unrelated cause for this individual’s elevated responses, their data were removed from the study prior to statistical analysis. Data recorded from the other two cats were included in the analysis.

#### 3.1.2 Bodyweight and food intake

All cats completing the study remained within 6% of their starting weight. The dosing of the supplement in a small amount of pate food was generally accepted by the cats. Five cats in total did not consume the whole bolus on all offered occasions. Two cats refused the full 8 g pate bolus (containing the supplement): one in the CBD group on one occasion and the other in the placebo group on 2 non-consecutive days. There were 15 partial refusals involving 5 g of pate bolus or less over the study, split between five cats (three in placebo and two in CBD group). The three cats in the placebo group accounted for 13 of the partial refusals.

#### 3.1.3 Postprandial plasma CBD time course after first dose and after 2 weeks of dosing

**Figure 1:**
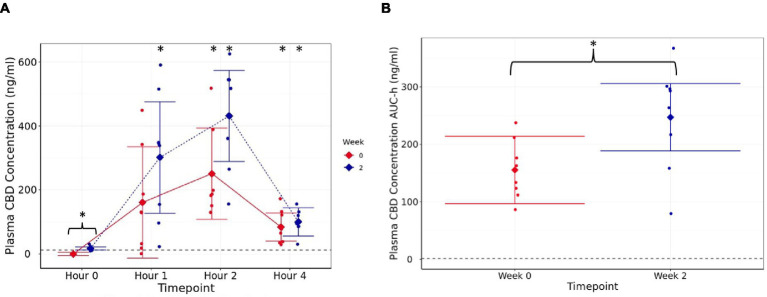
Plasma CBD concentration (ng/mL) **(A)** estimated means with 95% confidence intervals, at h 0 (fasted baseline), h 1, 2, and 4 measured at week 0 (d0, first supplement of 4 mg/kg BW, solid line) and week 2 (after 14 days of daily supplementation at 4 mg/kg BW, dotted line), **(B)** area under the curve (per hour) over the 4-h pharmacokinetic period for each timepoint (weeks). Dashed line shows limit of detection. * shows difference between the post-dose timepoint, and h 0 is statistically significant. The presence of a “}” in addition indicates the difference is between week 0 and week 2.

**Figure 2:**
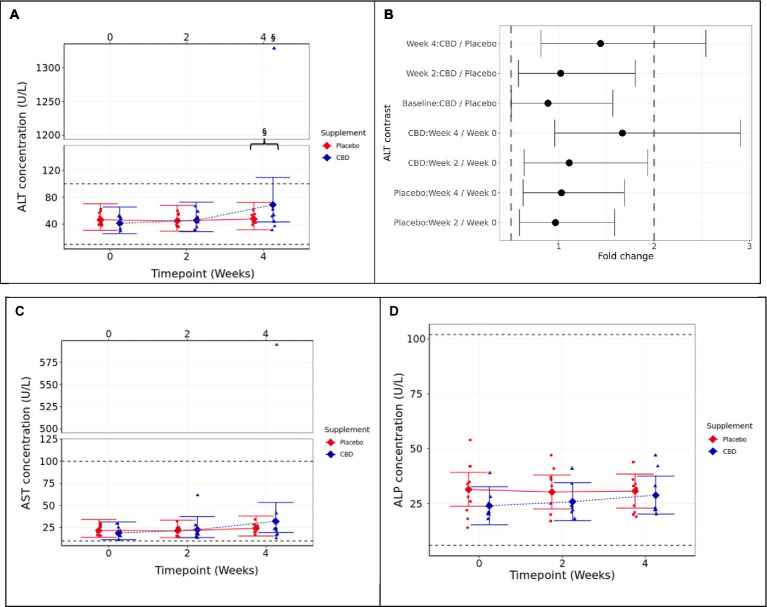
Means and 95% confidence intervals (C.I.) for fasted plasma measures over the 4-week study **(A)** ALT (U/L), **(B)** two one-sided test (TOST) fold change contrast plot, **(C)** AST (U/L), and **(D)** ALP (U/L). § indicates that equivalence between the timepoint and week 0 is not supported (*p* ≥ 0.05). The presence of a “}” in addition indicates non-equivalence between CBD treatment and placebo groups. Reference ranges included as dashed horizontal lines across the figure. TOST thresholds are shown with dashed vertical lines. Statistical equivalence is indicated by CIs falling entirely within these bounds (true for all contrasts except Week 4: CBD vs. Placebo, and CBD: Week 4 vs. 0). Statistical difference is indicated by CIs falling entirely to the left or right of 1 fold change. Liver health parameters.

At d0 (week 0) and week 2, the mean peak CBD concentration in the plasma occurred 2 h after dosing (*p* < 0.001; Figure 1A). The mean fasted (hour 0) CBD concentration was higher at week 2 than d0 (*p* < 0.001; Figure 2A), showing that CBD remains detectable in the plasma for up to 24 h after dosing. Although there were no significant differences postprandially (h 1, 2, and 4) between week 2 and d0 concentrations (*p* ≥ 0.12, Figure 2A), area under the curve data, collected over the 4 h sample period, were significantly higher at week 2 (246.9 ng-h/mL, 95% C.I: 188.4, 305.5) than at first dose (155.4 ng-h/mL, 95% C.I: 96.9, 214.0, *p* = 0.002, Figure 2B). Over the full 4 h time course, the AUC data were 621.7 (95% C.I: 387.5, 855.9) and 987.7 ng/mL (95% C.I: 753.5, 1222.0) for first dose and at week 2, respectively. At week 4, the mean fasted CBD concentration was 16.32 (range 10.8 to 28.03) ng/mL (data not shown).

#### 3.1.4 Liver health parameters

**Figure 3:**
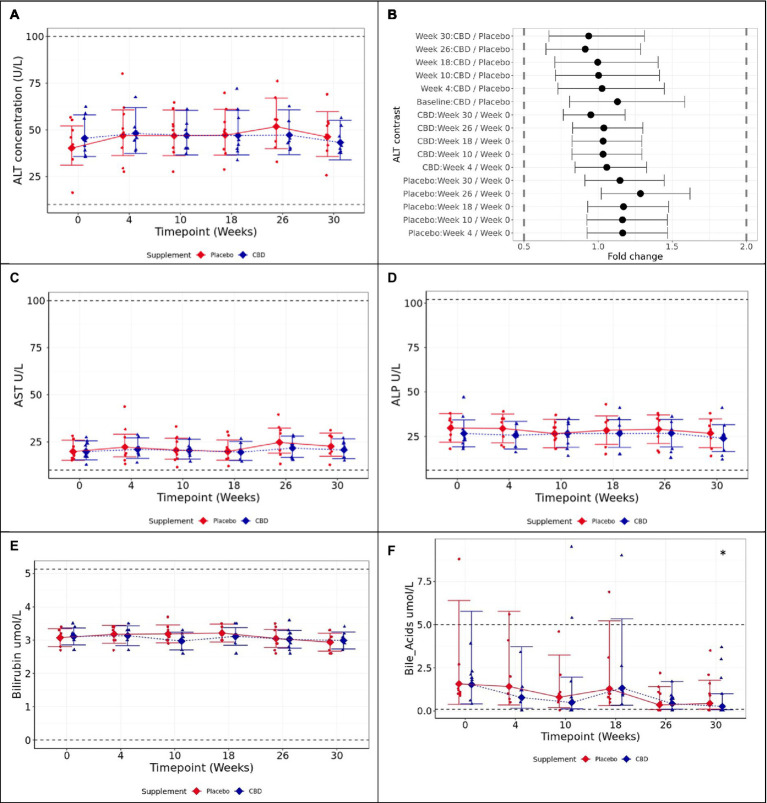
Means and 95% confidence intervals (C.I.) fasted plasma measures over the study **(A)** ALT (U/L), **(B)** two one-sided test (TOST) fold change contrast plot, **(C)** AST (U/L), **(D)** ALP (U/L), **(E)** bilirubin (μmol/L), and **(F)** bile acids (μmol/L). * shows difference between the timepoint, and week 0 is statistically significant (*p* < 0.05). Reference ranges included as dashed horizontal lines across the figure. TOST thresholds are shown with dashed vertical lines. Statistical equivalence is indicated by CIs falling entirely within these bounds (true for all contrasts). Statistical difference is indicated by CIs falling entirely to the left or right of 1 fold change.

For the CBD treatment group, two one-sided tests (TOSTs) failed to verify that the week 4 mean ALT concentration was below the upper 2-fold limit compared to either week 0 or the placebo control group (*p* ≥ 0.388; Figures 3A,B). The results indicate that cats in the CBD treatment group had higher mean ALT concentration at week 4 (1.667 fold change) compared to week 0 and also compared to placebo control cats (1.438 fold change).

Aspartate aminotransferase (AST) followed a similar trend to ALT data, and the CBD-treated cat that showed extremely high ALT also had a high AST value. However, this did not result in a significant difference between groups at week 4 (*p* = 0.81), nor between week 4 and baseline (*p* = 0.112; Figure 3C). Alkaline phosphatase (ALP) was also found not to significantly differ between treatment groups (*p* ≥ 0.447) or over time (*p* ≥ 0.062; Figure 3D). Bilirubin and fasted bile acids were not significantly different between groups (Supplementary data S1).

### 3.2 Long-term tolerance study

#### 3.2.1 Observations and/or removals from study

Three cats in total were removed from trial by week 10: one for consistently poor behavior during sampling (from week 4), one for high ALT (from week 4), and one for inappetence, high fasted bile acids, and high ALT (week 10). Veterinary consultation determined the presence of infection in both cats removed from trial for high ALT: one was from the placebo group and the other from the CBD group. Data from these three cats were incomplete and therefore excluded from statistical analysis.

#### 3.2.2 Bodyweight and food intake

All cats completing the study remained within 11% of their starting weight. The rate of bolus refusal was less than 1.5% of total offerings over the duration of the study. One cat in the CBD group fully refused the bolus on one occasion. In addition, five cats had partial refusals of the bolus: two in the placebo group (1 partial refusal each) and three cats from the CBD group. Two of the cats in the CBD group partially refused on 15 and 7 occasions, respectively, and the other cat partially refused on 1 occasion.

#### 3.2.3 Liver health parameters

**Figure 4:**
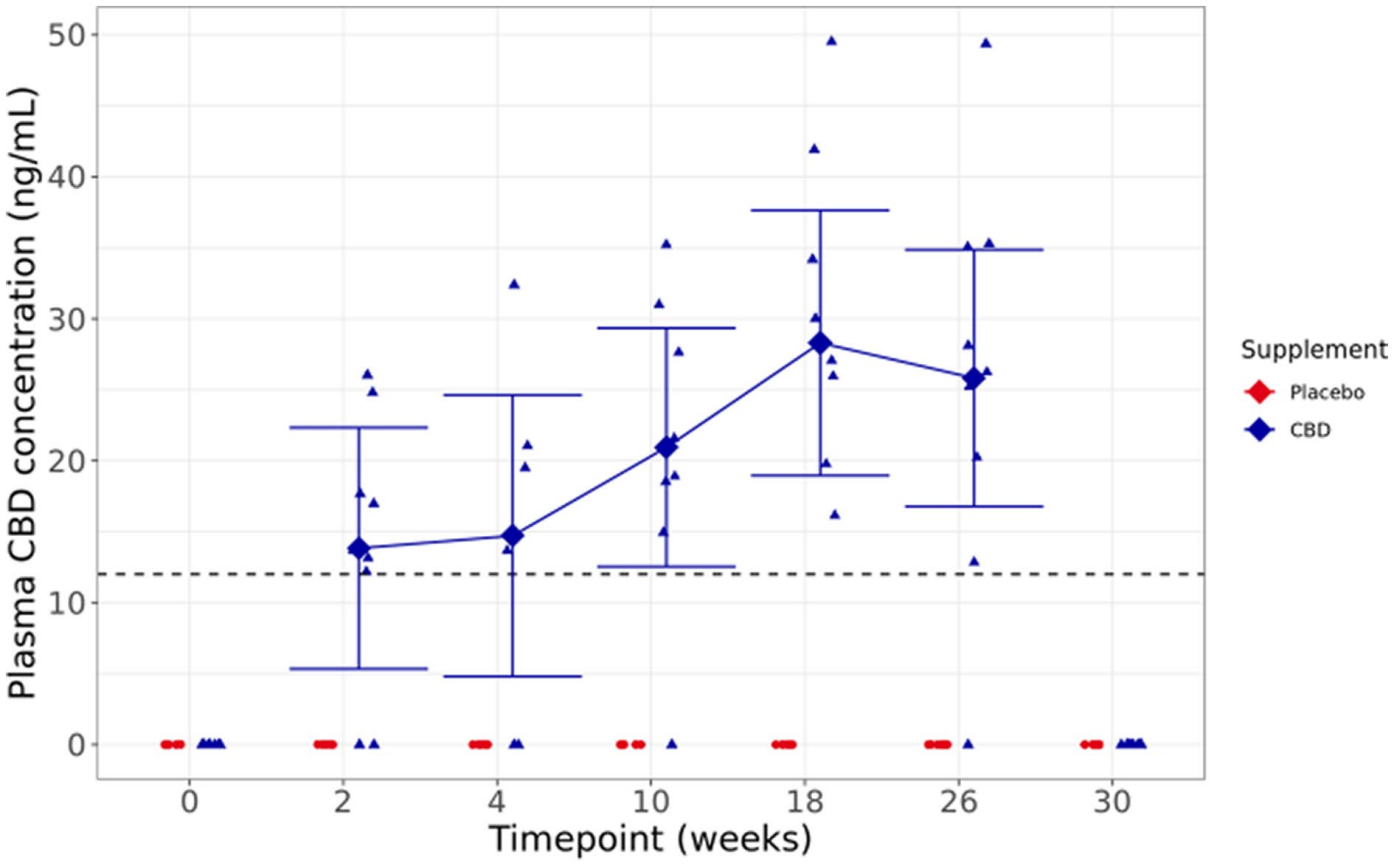
Means and within-model adjusted 95% confidence intervals for fasted plasma CBD concentration (ng/ml) over the study duration. Raw data points are plotted on the graph. Dashed line shows limit of detection.

Mean values of ALT, the primary study measure, were found to be statistically equivalent at 2-fold limits for each tested pairwise contrast, both between the placebo and CBD groups over the 26-week supplemented phase (*p* < 0.001), and by comparison with the respective week 0 baseline for each group (p < 0.001; Figures 4A,B). AST was found to be significantly increased at week 26 compared to baseline in placebo group cats, although all values remained within physiological reference range (*p* = 0.02; Figure 4C). ALP did not significantly differ over time within either group, or between groups at any timepoint (*p* ≥ 0.89; Figure 4D).

Bilirubin did not significantly differ between groups or over the course of the study in either group (*p* ≥ 0.725; Figure 4E). Fasted bile acids also did not significantly differ between CBD and placebo-treated groups across the study (*p* ≥ 0.997); however, CBD cats showed significantly reduced bile acid concentration at week 30 (washout) compared to baseline (*p* = 0.019; Figure 4F). For other biochemistry, hematology, bone-specific alkaline phosphatase (BALP), and carboxy-terminal telopeptide cross-link (CTx) data, there were no significant differences of clinical importance between the treatment groups. All measures were within normal reference ranges, with the exception of cholesterol, sodium:potassium ratio, mean platelet volume (MPV), and eosinophil count (Supplementary Tables S2, S3).

#### 3.2.4 CBD concentration

Visual inspection of fasted plasma CBD levels shows a broad range of concentrations, from below the limit of quantification to 49.52 ng/mL across the supplemented group, with a trend toward increased concentrations over the duration of the study. At the 30-week washout point, 4 weeks after dosing was completed, and there were no detectable levels of CBD in the plasma. One cat did not have detectable CBD concentrations at any sampled timepoint, despite full consumption of the bolus the day before each sampling (Figure 4).

Urine concentrations were all below the limit of detection (data not shown), while feces showed concentrations from 45.51 to 481.24 μg/g when a snapshot concentration was analyzed (data not shown).

## 4 Discussion

The aim of these studies in healthy cats was 2-fold: first, to evaluate the postprandial time course of plasma CBD concentrations over 4 hours following ingestion of an initial dose, and also after 2 weeks of daily supplementation at 4mg/kg BW; and second to demonstrate tolerance of daily supplementation of 4mg/kg BW over a 26-week period.

Peak mean CBD concentration in plasma was found at 2 h post-dose at both d0 (251 ng/mL) and week 2 (431 ng/mL), and CBD absorption and clearance rates were higher at week 2 compared to the first dose. This conclusion was inferred from the significant increase in AUC coupled with the lack of differences between the postprandial concentrations of plasma CBD. This timing broadly agrees with recent literature (14, 15). Deabold et al. (14) dosed six fasted cats with 2 mg/kg BW of a 50:50 CBD/CBDA mix in fish oil and reported a maximum concentration of 43 ng/mL at 2 h. Rozental et al. (25) also used a fasted protocol to evaluate a CBD isolate in sunflower oil, observing a maximum concentration at 2 h of 17.8 and 61.1 ng/mL for 2.5 and 5.0 mg/kg BW, respectively. Dosing of Epidiolex^®^ at 5 mg/kg BW to fed cats was found to increase the maximum concentration (465.3 ng/mL) and area under the curve (2650.0 h × ng/mL) data when compared to fasted dosing (269.0 ng/mL and 921.0 h × ng/mL, respectively) (18). In our study, the 2 h data of 251 ng/mL (95% CI: 108.7, 393.4) from cats fed a meal with their CBD dose are more similar to those reported by Wang et al. (15) for the first dose in their study at 282 ng/mL (±149.4), where the meal was fed 1 h after the CBD/CBDA paste offering. We observed an AUC upon first dose of 621.7 ng/mL (95% CI: 387.5, 855.9) over our 4 h time course compared to 908.5 ng/mL (±528.1) over 24 h in the literature (15), indicating that there is potentially a higher presence of CBD in the plasma after a dose of CBD/CBDA paste when compared to CBD in sunflower oil and that there is circulating CBD beyond the 4 h mark. When comparing these AUC values, it should be noted that pharmacokinetic software packages have been used by the other groups, likely employing a linear-log trapezoidal method, while our analysis employed a linear trapezoidal method which may overestimate the AUC (26). Given the comparatively short time frame (4 h) of our postprandial sampling, any impact of this overestimate should be limited. When comparing the literature, it is important to understand the compositions of the treatments being used as inclusion of several phytocompounds has been shown to alter the observed effects when compared to single phytocompounds. This observation, named the “entourage effect” by several publications (27–29), describes the potential for other compounds found in hemp, such as THC or CBG, to interact and possibly increase absorption of CBD (30). Both the U.S. Food and Drug Administration (FDA) and European Food Safety Authority (EFSA) have expressed concerns over the limitations of the current literature when it comes to interpreting safety due to the different preparations and extracts, varying concentrations of CBD or other cannabinoids, small sample sizes, and quality of the data (31, 32).

This is the first study in cats to report data on fasted plasma CBD concentrations using uniform dosing over a period of more than a week and after a washout period. Data were highly variable between individuals but showed overall increase in circulating concentrations up to week 18. This high variability between individual cats has been noted in the literature previously (14, 25). A recent theory describes body fat as a contributing factor, suggesting that with increased BCS there is a higher potential for CBD to be held in reservoir within the body (25). Our BCS data were insufficiently broad to assess this. Moreover, due to the semi-qualitative nature of this measurement and potential for assessor bias, the ability to confirm this via BCS is limited and alternative methods of assessing fat content should be considered. Similar trends in plasma CBD concentrations were seen in a comparable study performed in dogs, and additionally the routes of excretion, i.e., via the feces rather than urine, also appear to be consistent (11).

To evaluate whether chronic daily feeding of CBD was safely tolerated by the cats, both groups received regular veterinary examinations, as well as routine assessment of hematology, clinical biochemistry, and urinalysis. With the exception of cholesterol, sodium:potassium ratio, mean platelet volume (MPV), and eosinophil count, all group means for biochemistry and hematology analytes remained within published reference ranges throughout the 26-week study. Differences between groups or over time were transient and not deemed to be of clinical significance during the weekly and fortnightly veterinary reviews.

Across both studies, three cats experienced high ALT with concurrent inappetence and/or general lethargy. These cats were subsequently diagnosed, via abdominal ultrasound as well as blood biochemistry and complete blood count information, with suspected ascending cholangitis (an inflammation of the gall bladder and liver). One cat was in the placebo group and two in the CBD group. It is unknown whether the incidence of cholangitis was higher in the CBD group coincidentally or if the supplement (and/or involvement in the study) contributed through added pressure on the hepatic (and any linked) system and metabolic processes in these cats. The clinical opinion, however, was that CBD itself was unlikely to have caused the infection directly. Literature suggests that the prevalence of cholangitis in cats worldwide is common and cited as the second most common hepatic disease (33) across the four distinct forms of the condition: neutrophilic, lymphocytic, destructive, and chronic (34). The asymptomatic cat from study one was followed beyond study completion, no further complications were noted, and a return to normal range for ALT and AST occurred within 3 weeks. The changes in liver enzymes observed in specific individuals in the present studies, considered together with the variability of plasma CBD measurements, suggest that there are likely to be individual differences in the response of cats to multiple doses of CBD. It is not known whether there is a genetic basis for susceptibility to high ALT (or hypertransaminasemia). When evaluating adverse observations such as hypertransaminasemia potential drug interactions are the focus (35), however, during these studies we controlled access to any potential medications that could interact with CBD to minimize this risk. It is known that cats have a low capacity for hepatic glucuronidation which reduces the capacity for metabolism and excretion of several compounds including non-steroidal anti-inflammatories and CBD (16, 36). This may be a contributing factor to the differences between dog and cat responses to CBD; however, future work to address individual susceptibility could explore metabolomics of CBD absorption and excretion.

In conclusion, THC-free CBD fed at a dose of 4 mg/kg BW was absorbed into plasma and well tolerated when supplemented over 26 weeks in cats. However, caution should be applied, and veterinary checks recommended, if any history of liver issues is known or in the event of suspected concurrent infection. There is also further need for determining efficacy of CBD doses to improve our understanding of CBD and its use in cats.

## Data availability statement

The original contributions presented in the study are included in the article/Supplementary material, further inquiries can be directed to the corresponding author.

## Ethics statement

The animal study was approved by Waltham Animal Welfare and Ethical Review Body. The study was conducted in accordance with the local legislation and institutional requirements.

## Author contributions

JC: Conceptualization, Data curation, Supervision, Writing – original draft. RB: Data curation, Methodology, Project administration, Writing – review & editing. AB: Conceptualization, Writing – review & editing. ZE: Data curation, Formal analysis, Writing – original draft. CN: Formal analysis, Methodology, Writing – original draft. PW: Conceptualization, Supervision, Writing – review & editing. DL: Conceptualization, Supervision, Writing – review & editing. LH: Conceptualization, Supervision, Writing – review & editing.

## Supplementary material

SUPPLEMENTARY Table S1 Biochemistry, liver panel, BAP and CTX - mean estimates and 95% CIs.

SUPPLEMENTARY Table S2 Haematology - mean estimates and 95% CIs.

SUPPLEMENTARY FIGURE S1 Means and 95% confidence intervals (C.I.) fasted plasma measures over the 4 week study a) Bilirubin concentration (µmol/L) and b) bile acids (µmol/L). *Shows difference between the timepoint and week 0 is statistically significant (*p* < 0.05). Reference ranges included as dashed horizontal lines across the figure.
