# Supplementary material for: Healthy cats tolerate long-term daily feeding of Cannabidiol
Source: Front Vet Sci. 2024 Jan 24;10:1324622. doi: 10.3389/fvets.2023.1324622 (PMC10847353; doi:10.3389/fvets.2023.1324622)
Supplement: SUPPLEMENTARY Table S1 — Biochemistry, liver panel, BAP and CTX - mean estimates and 95% CIs. [file Table_1.DOCX]

S2 Biochemistry, liver panel, BAP and CTX - mean estimates and 95% CIs

| Parameter (95% CI) | Supplement group and Timepoint (weeks) | | | | | | | | | | | |
| --- | --- | --- | --- | --- | --- | --- | --- | --- | --- | --- | --- | --- |
|  | Placebo 0 | CBD 0 | Placebo 4 | CBD 4 | Placebo 10 | CBD 10 | Placebo 18 | CBD 18 | Placebo 26 | CBD 26 | Placebo 30 | CBD 30 |
| Total  Protein (g/L) | 68.77 | 69.21 | 68.36 | 66.91 | 68.04 | **65.81** | 69.17 | **66.57** | 70.48 | 68.8 | 69.7 | 69.19 |
|  | (65.46, 72.08) | (66.08, 72.33) | (65.04, 71.67) | (63.72, 70.11) | (64.72, 71.35) | **(62.61, 69.00)** | (65.85, 72.48) | **(63.38, 69.76)** | (67.17, 73.79) | (65.61, 71.99) | (66.38, 73.01) | (66.07, 72.31) |
| Albumin (g/L) | 31 | 31.39 | 31 | 30.91 | 31.54 | 30.65 | 31.77 | 30.93 | **33.29** | 32.12 | 31.59 | 31.31 |
|  | (28.7, 33.3) | (29.22, 33.56) | (28.7, 33.3) | (28.72, 33.1) | (29.23, 33.84) | (28.46, 32.83) | (29.47, 34.08) | (28.74, 33.11) | **(30.98, 35.59)** | (29.94, 34.31) | (29.28, 33.89) | (29.14, 33.48) |
| Glucose (mmol/L) | 4.62 | 4.62 | 4.64 | 4.52 | 4.78 | 4.38 | 4.49 | 4.61 | 4.57 | 4.61 | 4.69 | 4.49 |
|  | (4.25, 4.99) | (4.27, 4.97) | (4.28, 5.01) | (4.16, 4.88) | (4.42, 5.15) | (4.02, 4.73) | (4.12, 4.86) | (4.25, 4.97) | (4.2, 4.94) | (4.25, 4.96) | (4.33, 5.06) | (4.15, 4.84) |
| Inorganic  Phosphorus (mmol/L) | 1.18 | 1.18 | 1.24 | 1.22 | 1.29 | **1.3** | 1.25 | 1.27 | 1.21 | 1.23 | 1.26 | 1.24 |
|  | (1.07, 1.3) | (1.08, 1.29) | (1.12, 1.35) | (1.11, 1.33) | (1.17, 1.4) | **(1.19, 1.41)** | (1.13, 1.36) | (1.16, 1.38) | (1.1, 1.32) | (1.12, 1.34) | (1.15, 1.38) | (1.13, 1.34) |
| ALT (U/L) | 40.26 | 45.5 | 46.91 | 48.15 | 46.86 | 47 | 47.15 | 47.01 | 51.76 | 47.24 | 46.19 | 43.24 |
|  | (31.1, 52.1) | (35.68, 58.03) | (36.25, 60.72) | (37.44, 61.92) | (36.21, 60.65) | (36.54, 60.44) | (36.43, 61.02) | (36.58, 60.43) | (39.99, 66.99) | (36.74, 60.76) | (35.69, 59.78) | (33.9, 55.14) |
| AST (U/L) | 19.85 | 19.86 | 22.24 | 20.99 | 20.61 | 20.44 | 19.9 | 19.55 | **24.76** | 21.69 | 22.69 | 20.68 |
|  | (15.19, 25.93) | (15.44, 25.55) | (17.02, 29.05) | (16.23, 27.15) | (15.78, 26.93) | (15.81, 26.44) | (15.23, 26) | (15.13, 25.27) | **(18.96, 32.35)** | (16.77, 28.05) | (17.37, 29.65) | (16.07, 26.61) |
| Calcium (mmol/L) | 2.38 | 2.38 | 2.39 | 2.38 | 2.42 | 2.41 | 2.42 | 2.39 | **2.47** | 2.43 | 2.4 | 2.38 |
|  | (2.29, 2.47) | (2.3, 2.46) | (2.3, 2.48) | (2.29, 2.46) | (2.33, 2.51) | (2.32, 2.49) | (2.33, 2.5) | (2.3, 2.47) | **(2.39, 2.56)** | (2.35, 2.52) | (2.31, 2.49) | (2.3, 2.46) |
| Cholesterol (mmol/L) | 7.12 | 6.83 | 6.89 | 6.29 | 6.92 | 6.33 | 6.67 | **6.03** | 6.61 | 6.4 | 6.61 | 6.6 |
|  | (5.39, 8.84) | (5.21, 8.46) | (5.16, 8.61) | (4.66, 7.92) | (5.2, 8.65) | (4.7, 7.96) | (4.95, 8.4) | **(4.39, 7.66)** | (4.88, 8.33) | (4.77, 8.03) | (4.89, 8.34) | (4.97, 8.22) |
| Urea (mmol/L) | 8.88 | 8.87 | 8.56 | 8.51 | 9.15 | 9.16 | 8.85 | 8.9 | 8.88 | 8.58 | 8.54 | 8.66 |
|  | (8.16, 9.6) | (8.19, 9.55) | (7.84, 9.28) | (7.81, 9.21) | (8.43, 9.87) | (8.46, 9.86) | (8.13, 9.57) | (8.2, 9.59) | (8.16, 9.6) | (7.88, 9.28) | (7.82, 9.26) | (7.98, 9.34) |
| Magnesium (mmol/L) | 0.8 | 0.78 | 0.8 | 0.78 | 0.83 | 0.8 | 0.82 | 0.8 | 0.82 | 0.8 | 0.81 | 0.8 |
|  | (0.74, 0.87) | (0.72, 0.84) | (0.74, 0.87) | (0.71, 0.84) | (0.76, 0.9) | (0.73, 0.86) | (0.75, 0.89) | (0.74, 0.86) | (0.76, 0.89) | (0.74, 0.86) | (0.75, 0.88) | (0.74, 0.86) |
| Triglycerides (mmol/L) | 0.31 | 0.34 | 0.3 | 0.26 | 0.3 | 0.29 | 0.31 | 0.28 | 0.33 | 0.3 | 0.32 | 0.31 |
|  | (0.2, 0.42) | (0.23, 0.44) | (0.19, 0.42) | (0.15, 0.37) | (0.19, 0.42) | (0.18, 0.4) | (0.19, 0.43) | (0.16, 0.4) | (0.22, 0.45) | (0.19, 0.41) | (0.2, 0.43) | (0.2, 0.42) |
| ALP (U/L) | 29.75 | 26.67 | 29.5 | 25.66 | 26.63 | 26.66 | 28.5 | 26.61 | 29 | 26.78 | 26.75 | 24 |
|  | (21.72, 37.78) | (19.09, 34.24) | (21.47, 37.53) | (17.88, 33.44) | (18.59, 34.66) | (18.88, 34.44) | (20.47, 36.53) | (18.85, 34.38) | (20.97, 37.03) | (19, 34.57) | (18.72, 34.78) | (16.43, 31.57) |
| Creatinine (mmol/L) | 112.11 | 120.28 | 109.99 | 118.65 | 114.02 | 122.3 | **108.85^a^** | **124.48^a^** | 119.77 | 123.48 | 112.11 | 116.51 |
|  | (103.53, 120.69) | (112.18, 128.37) | (101.41, 118.57) | (110.17, 127.14) | (105.44, 122.6) | (113.82, 130.79) | **(99.25, 118.44)** | **(114.98, 133.98)** | (111.19, 128.35) | (115.00, 131.97) | (103.52, 120.69) | (108.42, 124.61) |
| Sodium (mmol/L) | 149.25 | 149.6 | 149.05 | 149.93 | 149.5 | 149.34 | 149.9 | 150.17 | 150.03 | **151.04** | **150.69** | 149.92 |
|  | (147.85, 150.65) | (148.28, 150.92) | (147.65, 150.45) | (148.56, 151.29) | (148.1, 150.9) | (147.97, 150.7) | (148.5, 151.3) | (148.81, 151.53) | (148.63, 151.42) | **(149.67, 152.4)** | **(149.29, 152.08)** | (148.61, 151.24) |
| Potassium (mmol/L) | 3.82 | 3.82 | 3.88 | 3.75 | 3.98 | 3.81 | 3.86 | 3.78 | 3.74 | 3.81 | 3.77 | 3.8 |
|  | (3.61, 4.03) | (3.63, 4.02) | (3.67, 4.09) | (3.54, 3.95) | (3.77, 4.18) | (3.6, 4.01) | (3.65, 4.07) | (3.58, 3.98) | (3.53, 3.95) | (3.61, 4.02) | (3.56, 3.98) | (3.6, 4) |
| Chloride (mmol/L) | 120.39 | 120.48 | 120.95 | 121.6 | 120.58 | 120.71 | 121.34 | 121.77 | 119.39 | 120.99 | 120.33 | 120.68 |
|  | (118.62, 122.15) | (118.81, 122.14) | (119.18, 122.72) | (119.84, 123.36) | (118.81, 122.34) | (118.95, 122.47) | (119.57, 123.1) | (120.01, 123.53) | (117.62, 121.15) | (119.22, 122.75) | (118.56, 122.09) | (119.01, 122.34) |
| Globulin (g/L) | 37.11 | 38.94 | 37.36 | 36.3 | 36.5 | 35.45 | 37.39 | **33.92** | 37.19 | 36.97 | 38.11 | 37.88 |
|  | (33.22, 41) | (35.1, 42.78) | (34.23, 40.49) | (33.23, 39.36) | (33.37, 39.63) | (32.39, 38.52) | (34.26, 40.52) | **(30.87, 36.98)** | (34.06, 40.32) | (33.91, 40.04) | (34.98, 41.24) | (34.93, 40.83) |
| AG ratio | 0.83 | 0.83 | 0.83 | 0.86 | 0.87 | 0.88 | 0.85 | 0.81 | 0.9 | 0.89 | 0.83 | 0.84 |
|  | (0.68, 0.99) | (0.67, 0.98) | (0.7, 0.96) | (0.74, 0.99) | (0.73, 1) | (0.75, 1) | (0.72, 0.99) | (0.68, 0.94) | (0.76, 1.03) | (0.76, 1.02) | (0.7, 0.96) | (0.71, 0.96) |
| Sodium-potassium  ratio | 40.12 | 39.23 | 38.65 | 40.08 | 37.68 | 39.26 | 38.91 | 39.82 | 40.24 | 39.65 | 40.12 | 39.56 |
|  | (37.32, 42.92) | (36.46, 41.99) | (36.41, 40.9) | (37.88, 42.27) | (35.44, 39.93) | (37.06, 41.46) | (36.67, 41.16) | (37.63, 42.01) | (38, 42.48) | (37.45, 41.85) | (37.88, 42.36) | (37.45, 41.67) |
| Bilirubin (µmol/L) | 3.08 | 3.11 | 3.18 | 3.13 | 3.19 | 2.97 | 3.21 | 3.11 | 3.05 | 3.02 | 2.94 | 2.99 |
|  | (2.81, 3.35) | (2.86, 3.37) | (2.91, 3.45) | (2.84, 3.43) | (2.92, 3.46) | (2.71, 3.24) | (2.94, 3.48) | (2.85, 3.38) | (2.78, 3.32) | (2.76, 3.29) | (2.67, 3.21) | (2.73, 3.24) |
| Bile  Acids (µmol/L) | 1.57 | 1.53 | 1.42 | 0.78 | 0.79 | 0.49 | 1.28 | 1.33 | 0.35 | 0.43 | 0.44 | **0.27** |
|  | (0.38, 6.40) | (0.41, 5.77) | (0.35, 5.77) | (0.16, 3.72) | (0.19, 3.24) | (0.12, 1.96) | (0.31, 5.22) | (0.33, 5.34) | (0.09, 1.42) | (0.11, 1.71) | (0.11, 1.78) | **(0.07, 1.00)** |
| BAP (U/L) | 10.25 | 10.45 | - | - | - | - | - | - | 11.61 | 11.92 | 11.48 | 10.47 |
|  | (7.43, 13.07) | (7.86, 13.04) |  |  |  |  |  |  | (8.79, 14.43) | (9.27, 14.57) | (8.73, 14.23) | (7.88, 13.06) |
| CTX (ng/mL) | 0.71 | 0.65 | - | - | - | - | - | - | 0.61 | 0.66 | 0.58 | 0.58 |
|  | (0.48, 0.94) | (0.44, 0.87) |  |  |  |  |  |  | (0.37, 0.84) | (0.45, 0.88) | (0.35, 0.8) | (0.36, 0.8) |

Bold numbers show significant difference (p<0.05). No superscript means difference is to week 0. Superscript means difference between groups at that timepoint.
