# Supplementary material for: Healthy cats tolerate long-term daily feeding of Cannabidiol
Source: Front Vet Sci. 2024 Jan 24;10:1324622. doi: 10.3389/fvets.2023.1324622 (PMC10847353; doi:10.3389/fvets.2023.1324622)
Supplement: SUPPLEMENTARY Table S2 — Haematology - mean estimates and 95% CIs. [file Table_2.DOCX]

S3 Haematology - mean estimates and 95% CIs

| Parameter  (95% CI) | Supplement group and Timepoint (weeks) | | | | | | | | | | | |
| --- | --- | --- | --- | --- | --- | --- | --- | --- | --- | --- | --- | --- |
|  | Placebo 0 | CBD 0 | Placebo 4 | CBD 4 | Placebo 10 | CBD 10 | Placebo 18 | CBD 18 | Placebo 26 | CBD 26 | Placebo 30 | CBD 30 |
| Red blood cell (M/µL) | 8.79 | 9.06 | 8.7 | 8.86 | 9.08 | 8.91 | 8.72 | 8.71 | 9.39 | 8.96 | 9.06 | 8.69 |
|  | (7.77, 9.81) | (8.1, 10.03) | (7.68, 9.73) | (7.84, 9.88) | (8.06, 10.1) | (7.93, 9.89) | (7.69, 9.74) | (7.73, 9.69) | (8.37, 10.41) | (7.98, 9.94) | (8.04, 10.09) | (7.73, 9.65) |
| Haemoglobin (g/dL) | 11.35 | 12.18 | 11.72 | 12.14 | 12.16 | 12.14 | 11.69 | 12.04 | **12.65** | 12.42 | 12.28 | 12.14 |
|  | (10.18, 12.52) | (11.07, 13.28) | (10.55, 12.89) | (10.89, 13.38) | (10.99, 13.33) | (11, 13.28) | (10.53, 12.86) | (10.91, 13.18) | **(11.48, 13.82)** | (11.28, 13.57) | (11.11, 13.44) | (11.04, 13.24) |
| Haematocrit (%) | 33.57 | 35.31 | 35.06 | 35.73 | 36.33 | 35.67 | 34.92 | 35.3 | **38.33** | 37.06 | **37.65** | 35.87 |
|  | (29.39, 37.75) | (31.37, 39.26) | (30.88, 39.24) | (31.39, 40.08) | (32.15, 40.51) | (31.62, 39.73) | (30.74, 39.1) | (31.25, 39.34) | **(34.15, 42.51)** | (33.01, 41.11) | **(33.47, 41.83)** | (31.93, 39.81) |
| Mean cell volume (fL) | 38.33 | 39.2 | **40.41** | 40.69 | **40.11** | 40.37 | **40.16** | 40.79 | **40.92** | **41.66** | **41.74** | **41.36** |
|  | (34.75, 41.92) | (35.82, 42.58) | **(36.82, 44)** | (37.22, 44.17) | **(36.52, 43.69)** | (36.96, 43.78) | **(36.58, 43.75)** | (37.39, 44.2) | **(37.34, 44.51)** | **(38.25, 45.07)** | **(38.15, 45.32)** | **(37.98, 44.75)** |
| Mean corpuscular haemoglobin (pg) | 13.01 | 13.56 | 13.55 | 13.84 | 13.44 | 13.77 | 13.18 | 13.96 | 13.54 | 14.03 | 13.63 | 14.04 |
|  | (11.74, 14.28) | (12.36, 14.76) | (12.28, 14.82) | (12.61, 15.08) | (12.16, 14.71) | (12.56, 14.98) | (11.9, 14.45) | (12.75, 15.17) | (12.27, 14.81) | (12.82, 15.24) | (12.36, 14.9) | (12.84, 15.24) |
| Mean corpuscular haemoglobin concentration (g/dL) | 33.99 | 34.54 | 33.54 | 34 | 33.53 | 34.07 | 33.56 | 34.16 | **33.12** | **33.62** | **32.68** | **33.89** |
|  | (32.64, 35.39) | (33.25, 35.89) | (32.21, 34.93) | (32.7, 35.35) | (32.2, 34.92) | (32.79, 35.41) | (32.22, 34.95) | (32.87, 35.49) | **(31.81, 34.49)** | **(32.36, 34.94)** | **(31.38, 34.03)** | **(32.62, 35.21)** |
| Red blood cell distribution width standard deviation (fL) | 30.46 | 30.51 | 30.61 | 30.6 | 29.9 | 29.62 | 29.93 | 29.99 | 31.29 | 30.11 | 30.92 | 30.61 |
|  | (28.65, 32.26) | (28.81, 32.21) | (28.8, 32.41) | (28.71, 32.5) | (28.1, 31.71) | (27.87, 31.37) | (28.13, 31.74) | (28.24, 31.74) | (29.48, 33.09) | (28.36, 31.87) | (29.11, 32.72) | (28.9, 32.31) |
| Red blood cell distribution width coefficient of variation (%) | 25.82 | 25.45 | **24.42** | 24.55 | 24.74 | 24.26 | **24.41** | 24.45 | 25.3 | **23.81** | **24.33** | **24.04** |
|  | (23.28, 28.37) | (23.04, 27.85) | **(21.88, 26.97)** | (22.09, 27.01) | (22.19, 27.29) | (21.84, 26.68) | **(21.86, 26.95)** | (22.03, 26.86) | (22.75, 27.85) | **(21.39, 26.23)** | **(21.78, 26.88)** | **(21.64, 26.44)** |
| Reticulocytes (K/µL) | 11.42 | 13.11 | 9.48 | 15.88 | 9.53 | 14.95 | 8.99 | 14.37 | 12.22 | 14.74 | 10.65 | 14.64 |
|  | (6.02, 16.82) | (8.02, 18.2) | (4.08, 14.88) | (9.93, 21.83) | (4.13, 14.92) | (9.63, 20.28) | (3.6, 14.39) | (9.06, 19.68) | (6.82, 17.62) | (9.42, 20.07) | (5.25, 16.05) | (9.55, 19.73) |
| Reticulocytes (%) | 0.13 | 0.15 | 0.11 | 0.18 | 0.11 | 0.17 | 0.1 | 0.17 | 0.13 | 0.16 | 0.12 | 0.17 |
|  | (0.06, 0.19) | (0.09, 0.21) | (0.05, 0.18) | (0.11, 0.25) | (0.04, 0.17) | (0.11, 0.23) | (0.04, 0.17) | (0.1, 0.23) | (0.07, 0.2) | (0.1, 0.23) | (0.06, 0.18) | (0.11, 0.23) |
| Immature reticulocyte fraction (%) | 39.96 | 39.47 | 44.75 | 38.24 | 44.61 | 41.11 | 39.93 | 33.55 | 39.28 | 38.57 | 36.72 | 39.62 |
|  | (24.72, 64.61) | (25.09, 62.08) | (27.68, 72.34) | (22.91, 63.82) | (27.59, 72.12) | (25.71, 65.74) | (24.7, 64.55) | (21, 53.58) | (24.3, 63.51) | (24.12, 61.67) | (22.71, 59.37) | (25.19, 62.32) |
| Low fluorescence ratio (%) | 56.51 | 57.63 | 50.71 | 54.81 | 51.21 | 55.65 | 56.89 | 62.72 | 57.3 | 57.96 | 57.75 | 55.48 |
|  | (35.91, 77.1) | (38.21, 77.04) | (30.12, 71.31) | (32.62, 77) | (30.62, 71.8) | (35.47, 75.83) | (36.3, 77.49) | (42.58, 82.85) | (36.71, 77.9) | (37.78, 78.14) | (37.16, 78.34) | (36.06, 74.9) |
| Medium fluorescence ratio (%) | 13.03 | 10.08 | 9.67 | 10.27 | 9.13 | 12.38 | 9.93 | 9.14 | 9.69 | 8.47 | 11.12 | 11.35 |
|  | (8.04, 18.03) | (5.37, 14.78) | (4.68, 14.66) | (4.52, 16.02) | (4.14, 14.13) | (7.39, 17.36) | (4.94, 14.92) | (4.16, 14.13) | (4.7, 14.69) | (3.48, 13.46) | (6.13, 16.11) | (6.64, 16.05) |
| High fluorescence ratio (%) | 27.46 | 27.14 | 34.79 | 26.06 | 34.71 | 27.21 | 28.56 | 23.59 | 28.86 | 28.35 | 24.71 | 26.07 |
|  | (14.28, 52.83) | (14.65, 50.3) | (18.09, 66.94) | (13.16, 51.6) | (18.04, 66.77) | (14.42, 51.35) | (14.84, 54.94) | (12.51, 44.45) | (15, 55.52) | (15.02, 53.51) | (12.85, 47.55) | (14.07, 48.31) |
| Platelet count (K/µL) | 242.35 | 272.89 | 229.67 | 238.34 | 299.13 | 237.52 | 197.69 | 255.88 | 244.65 | 265.61 | 246.63 | 198.54 |
|  | (105.82, 378.89) | (144.16, 401.61) | (93.13, 366.2) | (92.11, 384.57) | (162.59, 435.66) | (103.97, 371.08) | (61.16, 334.22) | (122.64, 389.12) | (108.11, 381.18) | (132.05, 399.16) | (110.09, 383.16) | (69.81, 327.26) |
| Mean platelet volume (fL) | 16.66 | 16.49 | 16.26 | 17.23 | 15.89 | 16.44 | 16.74 | 16.55 | 16.5 | 17.06 | 17.14 | 17.12 |
|  | (15.16, 18.31) | (15.09, 18.03) | (14.79, 17.87) | (15.6, 19.03) | (14.46, 17.46) | (15, 18.02) | (15.23, 18.4) | (15.1, 18.13) | (15.01, 18.13) | (15.56, 18.7) | (15.59, 18.84) | (15.66, 18.72) |
| PCT % | 0.4 | 0.45 | 0.37 | 0.4 | 0.47 | 0.39 | 0.33 | 0.42 | 0.4 | 0.44 | 0.4 | 0.34 |
|  | (0.18, 0.61) | (0.25, 0.65) | (0.15, 0.59) | (0.17, 0.63) | (0.26, 0.69) | (0.18, 0.6) | (0.11, 0.54) | (0.21, 0.64) | (0.18, 0.62) | (0.23, 0.66) | (0.18, 0.62) | (0.13, 0.54) |
| Total leukocyte count (K/µL) | 8.42 | 9.78 | 7.95 | 8.99 | 8.91 | 10.06 | 8.16 | 9.34 | 9.34 | 8.32 | 7.48 | 8.15 |
|  | (6.32, 11.21) | (7.47, 12.81) | (5.97, 10.59) | (6.7, 12.06) | (6.69, 11.87) | (7.63, 13.27) | (6.13, 10.86) | (7.09, 12.31) | (7.01, 12.43) | (6.31, 10.97) | (5.62, 9.96) | (6.22, 10.68) |
| Neutrophils (K/µL) | 3.95 | 4.6 | 3.74 | 3.99 | 4.11 | 4.44 | 4.06 | 4.09 | 4.76 | 3.84 | 4.07 | 3.8 |
|  | (2.44, 6.41) | (2.92, 7.26) | (2.3, 6.06) | (2.43, 6.56) | (2.53, 6.67) | (2.78, 7.09) | (2.51, 6.59) | (2.56, 6.51) | (2.94, 7.73) | (2.4, 6.12) | (2.51, 6.6) | (2.41, 5.99) |
| Lymphocytes (K/µL) | 2.97 | 3.55 | 2.74 | 3.44 | 3.11 | 3.7 | 2.62 | 3.29 | 2.81 | 3.13 | **1.94** | **2.76** |
|  | (1.81, 4.87) | (2.23, 5.66) | (1.67, 4.49) | (2.13, 5.56) | (1.9, 5.1) | (2.31, 5.92) | (1.6, 4.3) | (2.06, 5.27) | (1.71, 4.61) | (1.96, 5.01) | **(1.18, 3.19)** | **(1.73, 4.39)** |
| Monocytes (K/µL) | 0.25 | 0.22 | 0.24 | 0.23 | 0.27 | 0.27 | 0.26 | 0.24 | 0.29 | 0.23 | **0.3** | **0.27** |
|  | (0.17, 0.37) | (0.15, 0.32) | (0.16, 0.35) | (0.16, 0.33) | (0.19, 0.4) | (0.19, 0.39) | (0.18, 0.38) | (0.16, 0.34) | (0.2, 0.42) | (0.16, 0.33) | (0.21, 0.45) | (0.19, 0.39) |
| Eosinophils (K/µL) | 0.86 | 0.68 | 0.73 | 0.68 | 1 | 0.86 | 0.77 | 0.87 | 0.86 | 0.54 | 0.63 | 0.57 |
|  | (0.56, 1.16) | (0.39, 0.96) | (0.43, 1.03) | (0.37, 1) | (0.7, 1.3) | (0.57, 1.15) | (0.47, 1.07) | (0.58, 1.16) | (0.56, 1.16) | (0.25, 0.83) | (0.33, 0.93) | (0.29, 0.85) |
| Basophils (K/µL) | 0.07 | 0.06 | 0.06 | 0.05 | 0.07 | 0.07 | 0.07 | 0.06 | 0.06 | 0.05 | 0.04 | 0.04 |
|  | (0.04, 0.12) | (0.03, 0.1) | (0.03, 0.11) | (0.03, 0.1) | (0.04, 0.12) | (0.04, 0.13) | (0.04, 0.12) | (0.03, 0.11) | (0.03, 0.11) | (0.03, 0.08) | (0.02, 0.07) | (0.02, 0.08) |
| Neutrophils (%) | 48.1 | 49.72 | 48.92 | 46.28 | 47.27 | 46.35 | 51.33 | 46.73 | 53.38 | 48.21 | 56.76 | 49.42 |
|  | (33.58, 62.62) | (36.03, 63.41) | (34.4, 63.44) | (31.79, 60.78) | (32.75, 61.79) | (32.44, 60.26) | (36.81, 65.85) | (32.83, 60.62) | (38.86, 67.9) | (34.3, 62.12) | (42.24, 71.28) | (35.73, 63.11) |
| Lymphocytes (%) | 37.45 | 40.56 | 37.73 | 42.1 | 37.55 | 41.19 | 34.97 | 40.42 | 33.06 | 41.43 | 29.16 | 39.46 |
|  | (22, 52.9) | (26, 55.13) | (22.28, 53.18) | (26.98, 57.22) | (22.1, 53) | (26.48, 55.91) | (19.53, 50.42) | (25.71, 55.12) | (17.61, 48.51) | (26.71, 56.14) | (13.71, 44.61) | (24.89, 54.02) |
| Monocytes (%) | 2.99 | 2.28 | 3.02 | 2.53 | 3.09 | 2.71 | 3.13 | 2.53 | 3.07 | 2.82 | **4.06** | **3.38** |
|  | (2.18, 4.11) | (1.69, 3.08) | (2.19, 4.15) | (1.84, 3.49) | (2.25, 4.25) | (1.99, 3.67) | (2.28, 4.3) | (1.86, 3.43) | (2.23, 4.22) | (2.07, 3.82) | **(2.95, 5.58)** | **(2.5, 4.56)** |
| Eosinophils (%) | 10.43 | 6.59 | 9.38 | 8.09 | 11.12 | 8.55 | 9.56 | 9 | 9.62 | 6.72 | 8.99 | 6.91 |
|  | (6.83, 14.03) | (3.2, 9.98) | (5.78, 12.97) | (4.41, 11.77) | (7.52, 14.71) | (5.08, 12.02) | (5.96, 13.15) | (5.53, 12.46) | (6.02, 13.22) | (3.25, 10.2) | (5.39, 12.58) | (3.52, 10.31) |
| Basophils (%) | 0.79 | 0.55 | 0.76 | 0.56 | 0.75 | 0.63 | 0.78 | 0.66 | 0.68 | 0.53 | 0.53 | 0.53 |
|  | (0.41, 1.5) | (0.3, 1.02) | (0.4, 1.46) | (0.29, 1.07) | (0.39, 1.42) | (0.33, 1.21) | (0.41, 1.49) | (0.35, 1.25) | (0.35, 1.29) | (0.28, 0.99) | (0.28, 1.02) | (0.29, 0.98) |
| Reticulocyte haemoglobin (pg) | 15.2 | 15.69 | 14.95 | 16.31 | 16.06 | 15.65 | 15.67 | 16.31 | 15.57 | 16.08 | 15.74 | 15.83 |
|  | (13.27, 17.13) | (13.87, 17.51) | (13.02, 16.88) | (14.26, 18.37) | (14.13, 18) | (13.76, 17.53) | (13.74, 17.6) | (14.43, 18.19) | (13.64, 17.5) | (14.2, 17.97) | (13.81, 17.67) | (14.01, 17.65) |
| Red blood cell haemoglobin (%) | 14.06 | 14.57 | 14.49 | 14.98 | 14.23 | 14.71 | 14.38 | 15 | 14.41 | 15.05 | 14.45 | 14.61 |
|  | (12.85, 15.28) | (13.43, 15.72) | (13.27, 15.7) | (13.8, 16.16) | (13.01, 15.44) | (13.56, 15.87) | (13.17, 15.6) | (13.84, 16.15) | (13.2, 15.63) | (13.9, 16.21) | (13.23, 15.67) | (13.47, 15.76) |
