# Supplementary figures and images for: Healthy cats tolerate long-term daily feeding of Cannabidiol
Source: Front Vet Sci. 2024 Jan 24;10:1324622. doi: 10.3389/fvets.2023.1324622 (PMC10847353; doi:10.3389/fvets.2023.1324622)

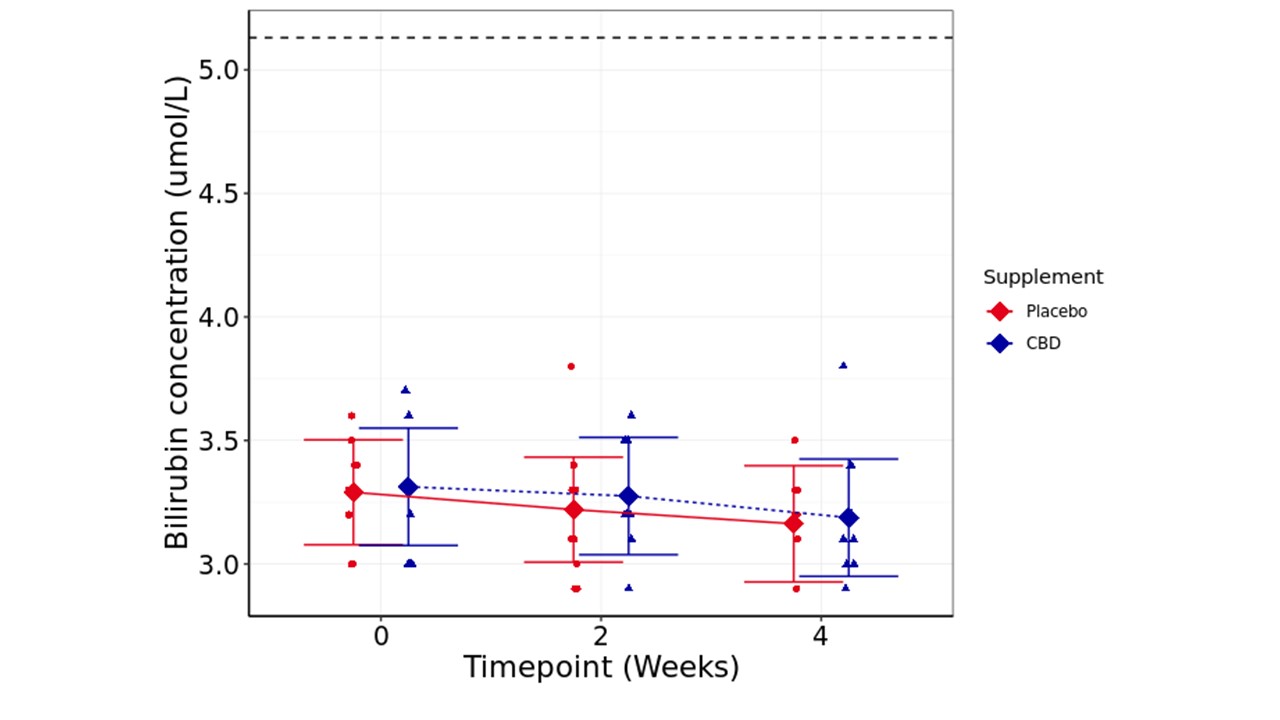

Supplement: SUPPLEMENTARY FIGURE S1 — Means and 95% confidence intervals (C.I.) fasted plasma measures over the 4 week study a) Bilirubin concentration (µmol/L) and b) bile acids (µmol/L). *Shows difference between the timepoint and week 0 is statistically significant (p < 0.05). Reference ranges included as dashed horizontal lines across the figure. [file Image_1.JPEG]

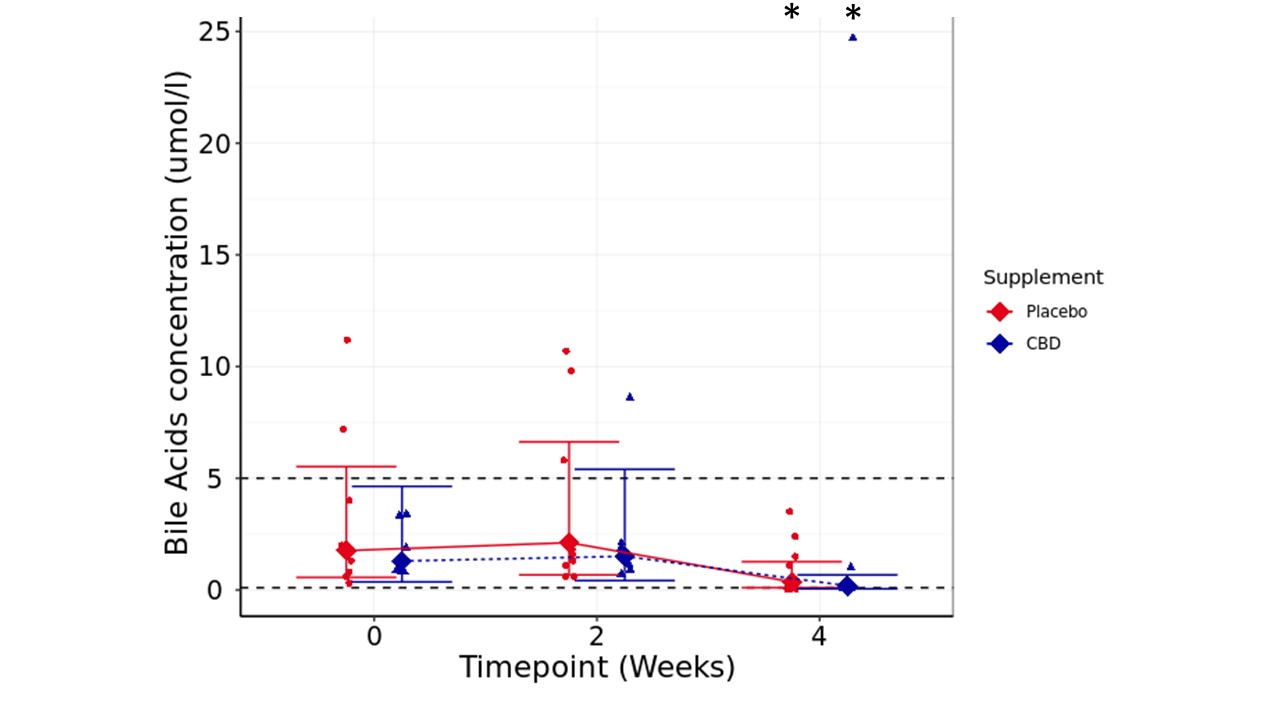

Supplement: Supplementary file 4 [file Image_2.JPEG]
